# Supplementary material for: Contact networks have small metric backbones that maintain community structure and are primary transmission subgraphs
Source: PLoS Comput Biol. 2023 Feb 23;19(2):e1010854. doi: 10.1371/journal.pcbi.1010854 (PMC9949650; doi:10.1371/journal.pcbi.1010854)
Supplement: Attachment — Submitted filename: letter-to-reviewers.pdf [file pcbi.1010854.s002.pdf]

Systems Science and Industrial Engineering Department
Thomas J. Watson College of Engineering and Applied Science
State University of New York at Binghamton
Binghamton, New York 13902

October 14, 2022

Jason A. Papin (Editor-in-Chief),
Nina Fefferman (Deputy Editor), and
Feng Fu (Associate Editor).
PLOS Computational Biology.

Dear editors and reviewers,

Thank you very much for your comments on our paper titled "The metric backbone preserves community structure and is a primary transmission subgraph in contact networks" under consideration at PLOS Computational Biology and for allowing us a reply. Below we address point-by-point each comment and indicate where in the main manuscript we made the corresponding changes.

Reviewer 1

> *This paper proposes the metric backbone of contact networks and shows the metric backbone preserves the community structures of the full contact networks and is a primary subgraph in epidemic transmission. I do not recommend that the paper be accepted until the problems raised below are addressed.*

We thank the reviewer for providing insightful comments about our paper and appreciate the chance to address each of the problems identified.

> *1. The main content of this paper is that the metric backbone can well preserve the community structure of the original network. However, the metric backbone has been defined in the reference [1] I want to know whether the metric backbone defined in this paper is the same as that in the references [1] If not, what is the specific difference? If so, this paper seems to only use numerical simulation to illustrate that the metric backbone can well maintain the community structure of the original network.*

We would like clarifying the important point that the metric backbone is an instance of the class of distance backbones defined in [1].The paper does not put forward the definition of the metric backbone as a novelty. Because the scope of PLOS CB is to "further our understanding of living systems at all scales—from molecules and cells, to patient populations and ecosystems—through the application of computational methods," our focus in the paper is on the novel application of the metric backbone to real-world contact networks, e.g., to understand how (shortest-path)

redundancy shapes the dynamics of epidemic transmission in human populations.

The metric backbone method has the potential for "wide adoption by a broad community of users" since it provides several important characteristics that are lacking in other network reduction methods (as shown in [1]): (1) It is algebraically principled, unique, deterministic, and null-model-free. (2) It typically results in fewer edges than other backbone methods. (3) It does not alter the value of edge weights. (4) It is guaranteed to preserve all shortest paths, nodes, and connectivity. The present paper, however, is not a methods paper. Rather, the goal is to demonstrate the use of the method to better characterize and understand the organization of social contact networks and the role of such organization in transmission dynamics.

Several novel observations ensue from our analysis of contact networks. Specifically, we discover that their metric backbone (a) is much smaller than the original networks, thus uncovering surprisingly large redundancy, (b) preserves community structure, both theoretically and experimentally, and (c) is a primary subgraph for simple transmission dynamics (or diffusion processes). This suggests that the organization of social contact networks is based on large amounts of shortest-path redundancy, which shapes epidemic spread in human populations.

To make sure we clarify the goal of the paper, we have updated the title and rewritten substantial portions of the abstract, introduction, and discussion sections, as shown in red edits therein.

> *2. Figure 2 needs to be redrawn. The function of metric backbone is the focus of this paper, so comparison of modules obtained with metric backbone $B(X)$ (not $D(x)$), and the threshold subgraph/ random subgraph.*

As we discuss in our response to comment 1, the focus of the paper is meant to be wider than the metric backbone. Still, we completely agree with the reviewer that it is very important that our results compare how community structure is uncovered on the metric backbone, $B(X)$, vis a vis same-size threshold and random subgraphs. Indeed that is the goal of Figure 2. We depict how each of the subgraphs—metric backbone and its same-size threshold and random subgraph comparison baselines—preserves the community structure detected on the full original graph, $D(X)$. Thus, a comparison with the $D(X)$ is necessary. The most important result that we want to emphasize with Figure 2 is that the baseline graphs break the community structure into smaller and mixed modules much more than the metric backbone does. This can be clearly seen by comparing the right-hand side of panels B, C, and D to one another. This way, we are directly comparing the backbone to the baselines as the reviewer requests.

An additional point of Figure 2 is to highlight that the metalabels do not necessarily provide a "gold standard" of modularity, and neither does the community structure of $D(X)$. Thus, panel A depicts a comparison between metalabels and community structure on $D(X)$ to help the reader appreciate their differences, as discussed in the text. But because the community structure detected on $D(X)$ or the metalabels cannot be taken as a gold standard, to strengthen our results we now report on using an alternative community detection method (Infomap) and simulated networks using stochastic block models (SBM) where we can know and control the true underlying community structure (see also response to reviewer 2).

In response to this comment, and to clarify what is conveyed in Figure 2, we have rewritten its caption and the respective discussion in section 3.1. Furthermore, in this section we have enhanced

the discussion of a lack of a modularity gold standard in the analyzed real-world social contact networks, and added several new paragraphs and a new Figure 3 with additional Infomap and SBM results that compare the metric backbone to the same-size threshold and random subgraph baselines. The new materials are highlighted in red throughout section 3.1 and respective supplemental materials (SM).

> *3. What I actually want to know is whether $B(X)$ preserves the community structure of the original network (the adjacency matrix instead of the distance graph $D(X)$). Similarly, compare the spread similarity of the disease on the adjacency matrix (not $D(X)$) and the metric backbone.*

The social contact networks are initially defined (in section 2) by a raw adjacency matrix, $R(X)$, whose edges tally the absolute number of measured contacts between two people. To compare networks across social contexts, typically, the raw adjacency matrix must be normalized. In our approach there are two versions of the normalized adjacency matrix: one represents a proximity, $P(X)$, and the other a distance relation, $D(X)$, among people in the network. These are given by equations 1 and 2, respectively. Crucially, $P(X)$ and $D(X)$ are isomorphic (as shown in [2]). This means that the metric backbone, and indeed all other network properties that depend on the proximity or distance weights, exist in both versions of the adjacency matrix (or either side of the isomorphism given by the map in eq. 2).

The reviewer is quite right, nonetheless, that methods like community detection and epidemic spread are defined for proximity weights, whereas shortest paths are defined for distance weights. We are very thankful to the reviewer for bringing up this issue that we had not explained sufficiently well in the previous version of the paper. In the new version, to clarify this issue, we have added a paragraph at the end of section 2 explaining the isomorphic relation and how and when we use the $P(X)$ and $D(X)$ adjacency matrices. Furthermore, throughout the paper, and especially in sections 3.1 and 3.2 and the figures therein, we clarify when we use $P(X)$ or $D(X)$, with new or edited text highlighted in red. Indeed, we have studied community structure and spreading dynamics using the proximity adjacency matrix (as these methods were designed to do), while shortest paths have been computed on the isomorphic distance adjacency matrix. This applies to all the real-world social networks, as well as the simulated networks of the newly added SBM simulations.

> *4. Nine different networks are mentioned in the text, but generally only one network is used as an example when numerical simulations illustrate experimental results. The experimental results are insufficient, and each numerical simulation needs to be performed on at least 4 networks without loss of generality.*

All simulations have been performed over 10 runs of the SI model starting from a random seed node and for 100 network realizations obtained via random edge removals for each value of χ. Thus, for each value of χ, 1000 simulations have been computed for each target backbone, threshold, and random subgraph. We have edited the caption of Figure 4 and section 3.2 on page 11 to make this clearer, with new or edited text highlighted in red.

The simulations summarized in Figure 4 were similarly conducted for all networks listed in Table 1, and are provided in supporting materials in detail. The text in section 3.2 (especially page 11) has been edited to make it clearer that our conclusions are supported by the results obtained for

every network—not just the French High School network shown in Figure 4—and are provided in SM.

5. In Figure 3, it is necessary to select different propagation parameters for experiments.

While we show results for only one value of β, this parameter only sets a global timescale for the spreading process. The values of the infection times $t_{1/2}$ and t_1 thus depend on β, but the values of $t_{1/2}$ and t_1 normalized by the values obtained on the full network do not. We have changed Figure 4 (and corresponding figures in SM for all other networks) to report on these normalized values that do not depend on β. Furthermore, we now mention in section 3.2, page 11, the fact that changing β does not impact the results, and show non-normalized values of $t_{1/2}$ and t_1 for two different values of β in Figure S8 in SM. All new or edited text is highlighted in red.

Minor Comments.
1. In Figure 1, the two pictures C and F seem to be just the rearrangement of the two pictures B and E (with gephi). Putting them all together is a bit repetitive, and it's easy to misunderstand that there are two different ways to generate subgraphs. Bur in fact there is only one.

Our goal with panels C and F in Figure 1 is to show that the metric backbone maintains and even highlights the community structure of the original network. Notice that on panels B and E, the nodes of the networks are rendered in exactly the same positions as those computed by the ForceAtlas2 algorithm when using every edge in the original networks. That is, nodes in panels B and E are in the exact same position as in panels A and D. What changes is the removal of the redundant edges. In contrast, the position of the nodes in panels C and F are completely recomputed with ForceAtlas2 but, crucially, using only the edges of the metric backbone.

What is striking, as we want to highlight with the figure, is precisely the apparent repetition the reviewer observes—especially in the case of the French High School network (panels A-C). The point is that the much sparser metric backbone graph, by itself, with the same rendering algorithm, leads to a community structure almost identical to the one rendered using the entire original network. In the case of the US high school (panels D-F), the rendering using only the backbone (panel F) actually better reveals the community structure computed using the entire original network (Panel D), even if sparsified to contain only the metric backbone (Panel E).

We have edited the caption of Figure 1 and the main text in section 3.1 to emphasize which networks are fed into ForceAtlas2 to visualize community structure in each panel of the figure. All new or edited text is highlighted in red.

2. Discussion is too long and needs to be shortened.

Given the various useful comments from both reviewers, we honestly find it very difficult to both be responsive and reduce the size of the discussion. Please reconsider the updated Discussion section for length.

3. Write out the specific generation method of threshold subgraph.

We have added a new subsection 3.2 to address this request.

Reviewer 2

> *The manuscript entitled as "The metric backbone preserves community structure and is a primary submission subgraph in contact networks" by Correia and others builds on a new network measure, called metric backbone, that was introduced by some of the authors in another publication [13]. This measure allows the identification of the superposition of shortest paths in weighted static networks. This is a very important problem, as its successful solution allows to use a reduced network representation of very dense structures to better explore their hidden structure and use them more efficiently for the modelling of ongoing dynamical processes.*
>
> *In this contribution, after introduction, the authors apply this new metric on nine real-world networks, which they obtain by aggregating temporal networks recorded in various settings and shared in the SocioPatterns project. As initial results they conclude that the identified backbones radically reduce the number of links while keeping the whole networks connected (e.g. without reducing their node sets). Subsequently they aim to demonstrate how much this method preserves the original (sometimes invisible) community structure of the original graphs as compared to some reference models. I found this part of the manuscript less convincing (a) because they base their claims on the success of one (modularity based) community detection method only (the Louvain method) and (b) although the identified backbone structures outperform the actual reference models, yet they consistently suggest many more community to be present, as compared to the original graph. In the third part of the manuscript they simulate an information spreading model on the constructed distance graph with different levels of reduction and compare their results to the corresponding reference models. Indeed their network outperforms the reference models. They show that even in the identified backbone the size of the reduced edge set is radically smaller, the speed of simulated spreading is not changing proportionally but provide comparable results to the dynamics simulated on the original network. I found this part of the manuscript more convincing.*
>
> *1. The paper is well written and scientifically sound. On the other hand, it provides only demonstrative results of an already established methodology. Moreover, although the simulated spreading process can be considered as the simplest example of an epidemic process, the contribution falls rather far from any direct biological application.*

We very much appreciate the thorough read, constructive comments, and understanding of our paper. Regarding what our work establishes and its novelty and interest, please first see our response to comment 1 from the first reviewer. Furthermore, while we would like for the metric backbone to be an established methodology, it has never been applied to the study of epidemic spread, where we believe it can have much utility.

First, in network epidemiology there is much interest in sparsification methods since they provide important computational gains, noise removal, and gains in understandability and explainability; this interest is highlighted by many publications on this topic, e.g. [3, 4, 5, 6]. But as we demonstrate and discuss in the paper, the metric backbone shows important advantages over existing

methods that have not hitherto been demonstrated or discussed in the study of epidemic spread. Please see the four main advantages listed in the response to comment 1 from the first reviewer.

Second, and perhaps more importantly, our work shows that the metric backbone reveals important characteristics of social contact networks and transmission dynamics that can lead to mitigation and intervention strategies for epidemics, which we also suggest. Again, please see the response to comment 1 from the first reviewer where we list three novel observations that ensue from our analysis of contact networks. In particular, the recognition that the organization of social contact networks is based on large amounts of shortest-path redundancy is novel. We show with empirical studies of nine distinct networks that this shortest-path redundancy shapes epidemic spread in human populations with simple transmission dynamics. Indeed, we show that typically very small backbone subgraphs are capable of preserving the entire distribution of shortest paths, which greatly facilitates the identification of a small set of edges that can be most effectively used for mitigation strategies. In contrast with same-size threshold and random graphs—which not only strongly alter the distribution of shortest paths but also break the underlying community structure much more and, importantly, most often decompose the original networks into disconnected components—the metric backbone analysis helps identify the small subset of edges whose removal leads to the largest impact on the shortest path distribution and thus on epidemic spread. In turn, these are suggested as effective targets for epidemic spread containment strategies. In the new version of the paper, especially in the abstract, introduction, and discussion sections, we clarify these points and what the methodology uncovers about epidemic spread on social contact networks, which is naturally of interest in biological applications. All the new text and edits are highlighted in red throughout the updated paper.

> *2. The title of the paper is very long and somewhat misleading, as the metric backbone is not precisely preserving the community structure of the original graph but gives a better approximation of it as compared to some reference models.*

We have revised the title of the paper to focus on what is newly uncovered about social contact networks, rather than the metric backbone methodology *per se*. Furthermore, instead of the term “preserve”, which may conjure “exactly preserving”, we use the term “maintain” which we feel is more general. Furthermore, we have enhanced the paper in a few distinct ways to better explain what we mean. First, it is important to understand that we have no access to a gold standard of what the true community structure of the real-world social contact networks is (see also our response to comment 2 from the first reviewer). We enhanced the discussion of this issue in section 3.1 and in the introduction and discussion sections.

To deal with the inability to know the true underlying community structure, in addition to the experimental results that compare the community structure of the original networks to that of the metric backbone and baseline same-size threshold and random sub graphs, we (a) highlight the theoretical results that ensue from the metric backbone construction, and (b) provide a new study with SBM.

Community structure refers to the identification of subsets of nodes that are more connected among themselves than to nodes in other subsets. In weighted graphs, this means subsets of nodes that are nearer one another while farther away from other subsets. Since the distance backbone preserves all shortest paths, it must preserve all intra- and inter-community shortest distances between every

pair of nodes. This is a theoretical feature that the baseline (and existing alternative sparsification) methods do not have. In this sense, it is true to say that the metric backbone best preserves all shortest distances of an underlying community structure: More specifically, it preserves the topology of the community structure when path distance is defined by the summation operation.

In addition to this important theoretical result, we also provide experimental evidence with synthetic networks where we can know and control the underlying community structure. This is done using SBM simulations now reported at the end of section 3.1 (with a new Figure 3) as well as in SM. While it would be too long to summarize the theoretical and experimental results in the title, given that both are expanded in the new version of the paper, we believe it is fair to state that metric backbones maintain community structure.

> *3. Page 3, Section 2, line 1: "Social contact networks are built by recording when individuals meet.": This is not a precise sentence. Social contact networks are recording with whom people meet. Only their temporal network representation is concerned by the question of "when". Since this contribution primarily concentrates on static structure, it would be better to rephrase this sentence.*

We agree and have rephrased this point.

> *4. Section 2: I missed from the introduction of the method the discussion of alternative shortest paths. Which shortest path the method is choosing between nodes if there are two fully equivalent shortest paths between them?*

The distance backbone methodology is based on edge, not path, properties. In the case of the metric backbone, all edges that break the triangle inequality via some indirect path (with length computed by summing all edges in path) are removed. Thus, the backbone comprises all edges, and only those edges, that do not break the triangle inequality—the metric edges. In other words, edges which directly link nodes x_i and x_j with distance weight d_{ij} remain in the backbone if and only if there are no shorter indirect paths between nodes x_i and x_j via any other set of nodes in the graph. Conversely, when an edge d_{ij} breaks the triangle inequality via other nodes x_{k_δ} (see eqs. 3 and 4), it is removed (or considered redundant), whether there exists only one or many shorter, alternative indirect paths. One does not have to choose between the several possible indirect shortest paths of same length, because it is the (semi-metric) edge that gets removed, not the alternative shortest paths (comprised of metric edges).

Due to this construction, if every edge on equivalent shortest paths between x_i and x_j happens to be metric, they all remain in the metric backbone, along with the possible paths they may form. This means that it is sometimes possible to reduce the metric backbone even further while preserving all shortest paths—a stricter transitive reduction which is beyond the scope of this paper but discussed in [1]. But in the edge-centered (not path-centered) construction of the metric backbone we define, the criterion is simply to identify the edges that obey the triangle inequality (eq. 4)—and remove those that break it (which can be further characterized by the semi-metric distortion of eq. 7). As a corollary of the construction, many alternative paths of the same length can exist between any two nodes in the backbone. To make this issue clearer, we have added a few sentences at the end of the paragraph defining metric and semi-metric edges and the triangle inequality in section 2.

5. Section 2: Although the original method is published in another paper, yet I missed some mention about the efficiency of the proposed method. What is the computational and memory complexity? How does the method scale? Can it be applied on very large or very dense weighted networks? This is not demonstrated in the paper as the analyzed real networks are rather small.

Because the method hinges on computing the All Pairs Shortest Paths (APSP) problem [7], the computational and memory complexity is that of the specific algorithm used to solve this problem, which is typically done in network science via Dijktra's algorithm [8]. The complexity of the fastest known algorithm for solving the APSP problem for weighted graphs is $\mathcal{O}(mn + n2logn)$, where n and m are the numbers of vertices and edges, respectively [2]. There are various approaches for solving the APSP problem, but all of them fall in the $\mathcal{O}(|X|^3)$ complexity range [7, 2]. We have added the computational complexity of the APSP problem to footnote 4 in section 2.

6. Section 3.1: Similar to the the title, I would tone down the title of this subsection.

We have changed the title of this subsection to "Metric backbone and community structure"

7. As demonstrated in Figure 1, although one would expect that a shortest path sub-graph is more tree like, the metric backbone structure contains surprisingly many triangles. This is probably because it operates with weighted shortest paths, yet it would help the reader if the authors could elaborate on this observation more.

As discussed in the response to comment 4, the metric backbone is constructed by evaluating edges, not shortest paths. It is quite distinct from a minimum spanning tree or similar constructs, as discussed in [1]. In particular, it can contain many equivalent (same length) shortest paths between the same two nodes. We have added a few sentences at the end of the paragraph defining metric and semi-metric edges and the triangle inequality in section 2 to emphasize this distinction. We refer the reader to [1] where many comparisons to other graph reductions are detailed since a detailed comparison is outside of the scope of this paper.

Please notice that for non-weighted graphs the metric backbone is the entire original graph because every edge is metric. This is discussed at the very start of the discussion section.

8. It would make the contribution stronger if the authors would test with more than one community detection method (using other paradigm as modularity), how much metric backbone "preserves community structure".

In this revised version, we have added Infomap as an additional community detection method, and the study of synthetic networks via SMB to section 3.1. Please see also our response to comment 2 (and our response to comment 2 from the first reviewer.) We have also enhanced the discussion of the visualization of community structure using the ForceAtlas2 algorithm (especially in regard to Figure 1 and similar figures in SM).

9. Table 2: Although the metric backbone outperforms the random and the thresholded networks in terms of identifying similar to real communities, yet the number of the

identified communities (17) is significantly larger than originally (10). This is one reason I think it is unfair to put forward as main result that this method "preserves communities". Actually it breaks them up considerably. The question is whether this is due to the sparsity of the backbone network or due to some bias the algorithm introduces?

This is related to comments 2, 6, and 8; see responses above. Clearly a big issue is not having access to the underlying "true" community structure, so we cannot always be certain that modularity detection on the original real-world networks uncovers the correct community structure. The new SBM analysis enhances our results by showing that even in low connectivity (high sparsity) scenarios, the metric backbone is superior in characterizing community structure. However, we essentially agree with the reviewer's assessment. Thus, we now emphasize in section 3.1 that our goal is simply to show that "the metric backbone presents a good compromise between preserving a substantially correct picture of the organization of contact networks while implementing sizeable and effective network sparsification." Furthermore, we have changed the title of the paper and subsection accordingly.

10. Figure 3: The meaning of the y axis is not well explained in the text or the caption. What "t (partial)" and "t (full network)" means in this context? Are the comparisons fair between networks which have different LCC sizes?

We now show times normalized by their values obtained when simulating the process on the full original network. We have clarified this point in the caption and the text in section 3.2. Moreover, we consider only networks where the largest connected component contains all the nodes.

11. Figure 3: The authors depict the y axis on a log scale and conclude that the spreading speed "remains much closer" to the original network as compared to the reference models. This is indeed true and it is well demonstrated visually, yet the logarithmic scale hides that there is a factor of 2 difference for the t_1 case and a factor of 4 difference for the $t_{1/2}$ cases as compared to $D(X)$. Regarding that the application of this methodology is meant for epidemic modeling, these differences are rather large. Although I find this result appealing and very interesting, this scaling should be discussed in the paper.

We have removed the logscale from the figure and separated the two distinct infection times into separate panels. Furthermore, we now provide normalized values of the times to half or full population infection, which yield the factors mentioned by the reviewer. The observed normalized ratios are clearer now, and even lower than they seemed in the previous figure: ≈ 1.6 for t_1 and ≈ 2.3 for $t_{1/2}$. These values are now explicitly discussed in the text of section 3.2, vis a vis those for the threshold and random same-size subgraph baselines—which are actually typically infinite at $\chi = 0\%$ since those baselines lead to disconnected graphs as their size approaches the size of the metric backbone.

12. Have the authors considered to compute the metric backbones of other types of networks, e.g. from biology?

Indeed, in [1] various biological networks ranging from brain connectome networks to gene regulatory networks are analyzed. Furthermore, a paper under review provides a large-scale study

of conserved gene regulation in spermatogenesis across species, which has revealed previously unknown genes involved in male infertility [9]. We are also working on a paper evaluating the metric backbone in the brain connectome of human cohorts across age groups. Another group has shown that the organization of semi-metric edges on the human connectome (i.e., those not on the metric backbone) can be used to discriminate autistic and depressive disorder patients[10] and, similarly, analysis of the functional reveals familial risk for psychotic disorder[11].

> *13. The authors completely missed to discuss the limitations of their results in the Discussion section.*

Thank you for pointing out this important oversight. We have expanded the last paragraph in the the discussion section to focus on limitations and future work.

Yours Sincerely,

Dr. Luis M. Rocha
rocha@binghamton.edu

References

1. Simas T et al. The distance backbone of complex networks. Journal of Complex Networks. 2021; 9(6):cnab021. DOI: 10.1093/comnet/cnab021
2. Simas T et al. Distance closures on complex networks. Network Science. 2015 Jun; 3(2):227–68. DOI: 10.1017/nws.2015.11
3. Bellingeri M et al. A comparative analysis of link removal strategies in real complex weighted networks. Scientific Reports. 2020; 10:3911. DOI: 10.1038/s41598-020-60298-7
4. Zhan XX et al. Information diffusion backbones in temporal networks. Scientific Reports. 2019; 9:6798. DOI: 10.1038/s41598-019-43029-5
5. Mercier AM et al. Effective Resistance for Pandemics: Mobility Network Sparsification for High-Fidelity Epidemic Simulation. 2022. DOI: 10.48550/arXiv.2111.02449. arXiv: 2111.02449

6. Serrano MÁ et al. Extracting the multiscale backbone of complex weighted networks. Proceedings of the National Academy of Sciences. 2009; 106:6483–8. DOI: 10.1073/pnas.0808904106
7. Zwick U. All Pairs Shortest Paths using Bridging Sets Retangular Matrix Multiplication. Journal of the ACM. 2002 May; 49:289–317
8. Dijkstra E. A note on two problems in connexion with graphs. Numerische mathematik. 1959; 1:269–71. DOI: 10.1007/BF01386390
9. Correia RB et al. The conserved transcriptional program of metazoan male germ cells uncovers ancient origins of human infertility. bioRxiv. 2022. DOI: 10.1101/2022.03.02.482557. eprint: https://www.biorxiv.org/content/early/2022/03/23/2022.03.02.482557.full.pdf. Available from: https://www.biorxiv.org/content/early/2022/03/23/2022.03.02.482557
10. Simas T et al. Commentary: Semi-Metric Topology of the Human Connectome: Sensitivity and Specificity to Autism and Major Depressive Disorder. Frontiers in Neuroscience. 2016 Aug; 10:353. DOI: 10.3389/fnins.2016.00353
11. Peeters S et al. Semi-metric analysis of the functional brain network: Relationship with familial risk for psychotic disorder. NeuroImage: Clinical. 2015; 9:607–16. DOI: 10.1016/j.nicl.2015.10.003
